# Uterine Broad Ligament Perivascular Epithelioid Cell Tumors (PEComa): A Case Report with 1-Year Follow-Up

**DOI:** 10.2174/0115734056397543251006112439

**Published:** 2025-10-15

**Authors:** Siying Zhang, Chunhong Yan, Feng Chen

**Affiliations:** 1 Department of Radiology, Zhejiang University School of Medicine First Affiliated Hospital,79 Qingchun Road, Hangzhou 310003, Zhejiang, China; 2 Department of Ultrasound, Zhejiang University School of Medicine First Affiliated Hospital,79 Qingchun Road, Hangzhou 310003, Zhejiang, China

**Keywords:** Uterine broad ligament, Perivascular Epithelioid Cell Neoplasm (PEComa), Computed tomography (CT), Magnetic resonance imaging (MRI), Tumor, PECs

## Abstract

**Introduction:**

This article presents a case of a patient with a broad ligament perivascular epithelioid cell tumor (PEComa), focusing on the analysis of its imaging features in CT and MRI to enhance understanding and awareness of this rare tumor.

**Case Presentation:**

This article reports a case of a 27-year-old married woman who was found to have a pelvic mass two years ago. After detailed examination at our hospital, imaging studies, including enhanced CT and MRI, revealed a cystic-solid lesion in the left adnexal area, with preoperative considerations of ovarian cystadenoma or uterine leiomyoma. She was referred to a specialized obstetrics and gynecology hospital for surgery, and the postoperative pathology was diagnosed as PEComa. She has been undergoing regular follow-up at our hospital post-surgery. One year after the operation, her laboratory tests showed no significant abnormalities, and imaging studies did not reveal any signs of metastasis.

**Conclusion:**

Uterine broad ligament PEComa is a rare tumor, and accurate imaging features and classification criteria can aid in improving preoperative diagnosis. A deeper understanding of the clinical and imaging characteristics of this rare disease is significant for enhancing diagnostic accuracy and treatment outcomes.

## 1 INTRODUCTION

Uterine broad ligament perivascular epithelioid cell tumor (PEComa) is a rare type of mesenchymal tumor that arises from perivascular epithelioid cells (PECs). The concept of PECs was first introduced in 1943 by Apitz *et al*., who described these cells as “abnormal myoblasts” found in renal angiomyolipomas [1]. Subsequently, in 1992, Bonetti *et al*. coined the term “perivascular epithelioid” to more accurately define this unique cell type, which is characterized by distinct morphological and immunohistochemical features, as well as a specific perivascular distribution [1, 2]. As research on PEComa has progressed, the World Health Organization (WHO) has classified these tumors as “mesenchymal tumors composed of histologically and immunohistochemically distinctive perivascular epithelioid cells” [3].

PEComas can occur in various anatomical sites, including soft tissues and the reproductive system, with an increasing number of reports in the field of gynecology in recent years [4, 5]. However, literature regarding the imaging features of uterine-related PEComa cases is relatively scarce, particularly studies focusing on uterine broad ligament PEComa are almost nonexistent.

Therefore, this article aims to present the specific case of a patient with uterine broad ligament PEComa, focusing on the analysis of its manifestations in imaging studies, including computed tomography (CT) and magnetic resonance imaging (MRI). By comprehensively analyzing these imaging features, we hope to enhance the understanding and awareness of this rare tumor.

## 2 CASE PRESENTATION

A 27-year-old married female patient presented for examination due to the discovery of a cystic lesion in the left adnexa, with a history of dysmenorrhea. After comprehensive examinations at our hospital, she was referred to a specialized obstetrics and gynecology hospital for surgical intervention. She has been undergoing regular follow-up at our hospital postoperatively. This research received approval from our hospital's ethics committee. All procedures carried out in this study adhered to the ethical guidelines set forth in the Declaration of Helsinki (revised in 2013). The patient provided written informed consent for the publication of this case report and the related images. A copy of the consent form is available for review by the editorial office of this journal.

### 2.1 Radiological Examination

**Fig. (1):**
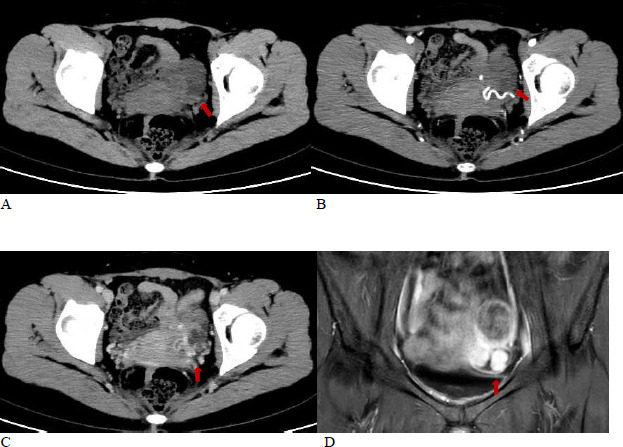
**A**. An irregular low-density mass can be seen around the left adnexa, with indistinct boundaries involving the uterus, measuring approximately 4.4 × 5.0 cm. **B**. In the arterial phase of the contrast-enhanced scan, there was mild to moderate enhancement observed in part of the lesion. **C**,**D**. In the venous phase of the contrast-enhanced scan, the solid part of the lesion showed further enhancement, presenting as significant enhancement.

The patient underwent enhanced CT and MRI examinations. The enhanced CT showed an irregular low-density mass in the left adnexa, with indistinct boundaries with the uterus, measuring approximately 4.4 × 5.0 cm (Fig. **1**). During the arterial phase post-enhancement, there was mild to moderate enhancement, and in the venous phase, there was significant enhancement. In the right adnexa, a slightly hyperdense lesion was noted, measuring about 1.4 × 1.6 cm, which showed no enhancement after contrast administration, suggestive of an endometriotic cyst.

**Fig. (2):**
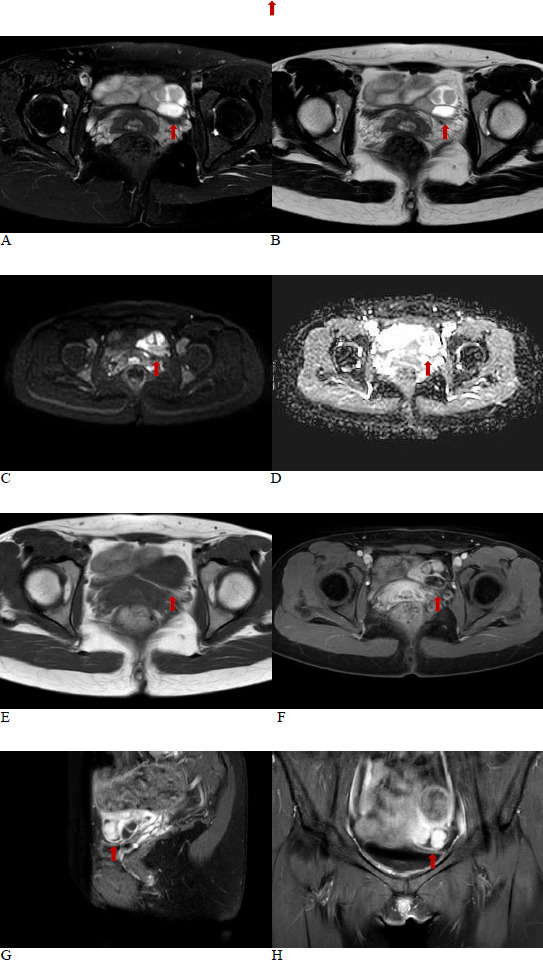
(**A**,**B**). Around the left adnexa, a cystic-solid lesion was observed, which showed heterogeneous high signal intensity on T2WI. The fat-suppressed T2WI sequence did not show any reduction, suggesting the absence of fat components. The left ovary was visible, with indistinct margins between it and the lesion. (**C**,**D**). The lesion showed high signal intensity in the solid component on diffusion-weighted imaging (DWI), and the ADC signal was reduced, indicating restricted diffusion. (**E**,**H**). The lesion showed iso-signal intensity on T1-weighted imaging (T1WI), and after enhancement, the solid component exhibited significant enhancement.

The enhanced MRI revealed a cystic-solid lesion in the left adnexa, displaying isointensity on T1-weighted imaging (T1WI) and heterogeneous hyperintensity on T2-weighted imaging (T2WI) (Fig. **2**). The solid component showed restricted diffusion on diffusion-weighted imaging (DWI) and significant enhancement post-contrast. The left ovary was visible, with unclear boundaries with the lesion, suggestive of an ovarian cystadenoma. In the right adnexa, there was an oval abnormal signal lesion, showing slightly hyperintense signal on T1WI, hyperintense signal on T2WI, with restricted diffusion on DWI, and no significant enhancement after contrast administration, suggestive of an endometriotic cyst.

### 2.2 Laboratory Tests and other Related Examinations

The results of laboratory tests and other related examinations showed no significant abnormalities in the complete blood count, coagulation profile, biochemical tests, hepatitis B panel, hepatitis series, sexually transmitted disease (STD) screening, tumor markers, and electrocardiogram (ECG), all of which were within normal ranges.

### 2.3 Surgical Findings

During the laparoscopic pelvic mass excision under general anesthesia, it was observed that the uterus was in a normal position and size, with a smooth surface and moderate texture, clearly delineated from surrounding tissues. The left parauterine tissue was notably thickened, within which a faintly visible purple-blue multilocular soft mass was observed, measuring approximately 5.5 x 4.5 x 5 cm. The left ovary appeared slightly smaller and firmer, with no abnormalities on its surface. The right ovary was also slightly smaller and firm, with a faintly palpable cyst measuring approximately 1.8 × 1.5cm. The bilateral fallopian tubes were soft in texture, with a normal shape, and the fimbrial mucosa was visible, along with the rectouterine pouch.

### 2.4 Pathological Results

**Fig. (3):**
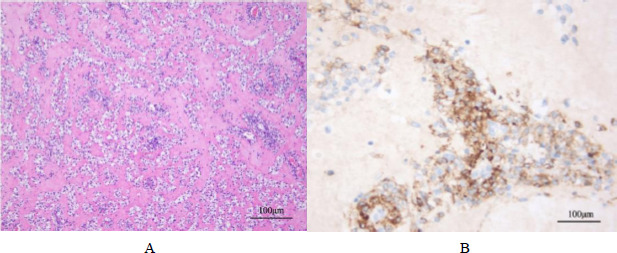
(**A**). Under low magnification, tumor cells exhibited diffuse growth, with a densely fibrotic stroma widely distributed, intermingling with the tumor cells. Under high magnification, tumor cells appeared epithelial-like, with abundant, clear cytoplasm. The nuclei were round or oval, with fine chromatin and visible nucleoli. No necrosis or mitotic figures were observed. (**B**). Imunmohistochemical results: Ki-67 (5% +), SMA (+), Desmin (+), CD34 (vascular +), P16 (focal +), HMB45 (partial +), Melan A (focal +), Cathepsin K (+), CD34 (vascular +), MyoD1 (plasma +).

Under low-power microscopy, tumor cells exhibited diffuse growth, with a widely distributed, sclerotic, fibrous stroma interwoven among the tumor cells. Under high-power microscopy, the tumor cells appeared epithelial-like, with abundant, clear cytoplasm (Fig. **3**). The nuclei were round or oval, exhibiting finely textured chromatin and visible nucleoli. No necrosis or mitotic figures were observed.

Immunohistochemical results were as follows: CK8/18 (-), Ki-67 (5% +), ALK (-), SMA (+), Desmin (+), CD34 (vascular +), S-100 (-), P16 (focal +), SYN (-), HMB45 (partial +), Melan A (focal +), Cathepsin K (+), CD34 (vascular +), MyoD1 (plasma +), and Myogenin (-).

Considering the morphology and immunohistochemical results, the diagnosis was consistent with a sclerosing PEComa.

The right ovary showed a benign cyst with hemosiderin deposition, with no overlying epithelium observed, and surrounding fibromatous proliferation, suggesting the possibility of an endometriotic cyst.

### 2.5 Follow-up

During the 1-year follow-up, the patient's laboratory tests revealed no significant abnormalities. Imaging studies showed no signs of metastasis.

## 3 DISCUSSION

Uterine broad ligament PEComa is a relatively rare tumor in clinical gynecology, primarily seen in adult women, typically in the 5^th^ to 7^th^ decades of life [6, 7]. Currently, numerous literature reports exist on the imaging characteristics of PEComa in various sites, including the liver, kidney, and lung [8-10]. However, studies on uterine broad ligament PEComa have primarily focused on pathological findings [11]. There is currently no literature specifically discussing the imaging features of uterine broad ligament PEComa.

Uterine broad ligament PEComa, regardless of its growth pattern, typically displays variable degrees of stromal hyalinization [1]. In some instances, this hyalinization is so pronounced that the epithelioid cells appear to be embedded within a hyalinized-fibrotic stroma. Uterine PEComas may be well-circumscribed, partially circumscribed, or show diffuse myometrial infiltration. The vascularization of PEComas is also characteristic, typically forming a network of small vessels distributed throughout the tumor.

Uterine broad ligament PEComa exhibits a tendency for local recurrence and may develop distant metastases, most frequently in the lungs. Metastasis can occur late, sometimes manifesting up to seven years after curative surgery [12].

Surgery is the primary treatment of choice, with adjuvant therapies typically reserved for high-risk cases. A subset of malignant gynecological PEComas exhibits aggressive behavior, characterized histologically by a high mitotic rate and multifocal necrosis [13]. While necrosis is associated with radioresistance, the high mitotic activity and rich vascularization of these tumors suggest heightened cellular sensitivity to radiation, supporting the consideration of radiotherapy for PEComas. However, the role of radiotherapy in the management of PEComas remains to be fully elucidated and necessitates further investigation.

In this case, the tumor was located around the left adnexa, with unclear boundaries involving the uterus and left ovary, presenting as a cystic-solid lesion, where the solid component showed enhancement and restricted diffusion. Differential diagnoses prior to surgery included ovarian origin cystadenoma and uterine leiomyoma. The density, signal, and enhancement characteristics of uterine leiomyoma were found to be similar to those of the uterine myometrium, and there was no significant enhancement noted in the necrotic and cystic areas on enhanced scans [14]. Uterine leiomyoma may also present as cystic-dominant solid masses. However, the solid component typically retains the imaging characteristics of uterine leiomyoma, thus appearing similar to the uterine myometrium on diffusion-weighted imaging (DWI). In this case, the solid portion on the DWI sequence showed higher signals than the myometrium, serving as a point of differentiation.

Ovarian cystadenomas are typically located within the ovaries; in this case, the left ovary was involved. Ovarian cystadenomas usually manifest as multilocular cystic lesions with multiple septations and may present with wall nodules [15]. In this case, the septations were not prominent, providing important clues for differential diagnosis.

Additionally, the patient experienced dysmenorrhea, which may be related to the endometriotic cyst in the right ovary, further complicating clinical evaluation.

It is noteworthy that the recent classification criteria for PEComa proposed by Folpe *et al*. provide important references for tumor risk assessment [1, 6, 16]. These criteria categorize high-risk features into six items, including tumor size ≥5 cm, infiltrative growth pattern, high nuclear grade, mitotic rate >1 per high-power field (HPF), necrosis, and vascular invasion. According to these criteria, PEComa can be classified into three categories [1, 6]: benign, uncertain malignant potential, and malignant. “Benign” tumors are defined as those without typical malignant features, while “uncertain malignant potential” tumors exhibit only a single histological feature, such as nuclear atypia, multinucleated giant cells, or a tumor size of ≥5 cm; “malignant” tumors demonstrate two or more abnormal features. These criteria are significantly associated with the invasive pathological behavior of the tumors.

Therefore, early accurate diagnosis of uterine broad ligament PEComa can significantly improve patient prognosis, reduce the risk of disease progression, and provide a basis for effective treatment plans.

## 4 STUDY LIMITATIONS

The single-case design limits generalizability, and the absence of comparative analysis with similar tumors restricts the ability to draw broader conclusions. A longer follow-up period and larger cohorts are needed to validate imaging features and prognostic factors.

## CONCLUSION

Uterine broad ligament PEComa is a rare tumor with nonspecific imaging features, making preoperative diagnosis challenging. This case highlights the importance of combining CT and MRI findings with pathological and immuno-histochemical analysis for accurate diagnosis. The tumor's cystic-solid appearance, enhancement patterns, and restricted diffusion on DWI provide key diagnostic clues; however, differentiation from ovarian cystadenoma and leiomyoma remains challenging.

The study underscores the need for larger, multicenter studies to establish definitive imaging criteria and improve preoperative assessment. While the patient showed no recurrence or metastasis at one-year follow-up, extended monitoring is essential to evaluate long-term outcomes, given the tumor's potential for late metastasis. These findings contribute to the limited literature on uterine broad ligament PEComa, emphasizing the role of multidisciplinary collaboration in managing this rare entity.

## Data Availability

The data of current study are available from corresponding author, [F.C], on a reasonable request.

## LIST OF ABBREVIATIONS

PEComa: Perivascular Epithelioid Cell Neoplasm
CT: Computed Tomography
MRI: Magnetic Resonance Imaging
DWI: Diffusion-Weighted Imaging
HPF: High-Power Field
WHO: World Health Organization
ECG: Electrocardiogram
STD: Sexually Transmitted Disease

## STANDARDS OF REPORTING

CARE guidelines were followed.
